# An extended conformation of SARS-CoV-2 main protease reveals allosteric targets

**DOI:** 10.1073/pnas.2120913119

**Published:** 2022-03-24

**Authors:** Zengchao Sun, Lu Wang, Xiyang Li, Chengpeng Fan, Jianfeng Xu, Zhenzhong Shi, Huarui Qiao, Zhongyun Lan, Xin Zhang, Lingyun Li, Xin Zhou, Yong Geng

**Affiliations:** ^a^The Chinese Academy of Sciences Key Laboratory of Receptor Research, State Key Laboratory of Drug Research, Shanghai Institute of Materia Medica, Chinese Academy of Sciences, Shanghai 201203, China;; ^b^Department of Biopharmaceutics, College of Food Science and Technology, Shanghai Ocean University, Shanghai 201306, China;; ^c^School of Basic Medical Sciences, Wuhan University, Wuhan 430071, China;; ^d^University of Chinese Academy of Sciences, Beijing 100049, China

**Keywords:** SARS-CoV-2 main protease, nanobody, M^pro^ extended conformation, M^pro^ compact conformation

## Abstract

The coronavirus main protease (M^pro^) is required for viral replication and has enzymatical activity as a homodimer. Thus, targeting its dimerization is an effective strategy for developing allosteric inhibitors to suppress mutation escape. In this study, we obtained the extended conformation of the native monomer of severe acute respiratory syndrome coronavirus 2 (SARS-CoV-2) M^pro^ by trapping it with nanobodies, and found that the catalytic domain and the helix domain dissociate, revealing allosteric targets. We also found another state, a compact conformation, similar to the dimeric form. Our data support that the M^pro^ may be in equilibrium among the monomeric extended conformation as the precursor of all other states, the compact conformation as the intermediate state, and the dimeric conformation as the active state. We designed an innovative Nanoluc Binary Technology (NanoBiT)-based high-throughput allosteric inhibitor assay based on the rearranged conformation. In addition, we identified a set of allosteric inhibitory nanobodies against M^pro^, one of which is also a competitive inhibitor of M^pro^. Our results provide insight into the maturation of the coronavirus M^pro^ and a way to develop anticoronaviral drugs through targeting the folding process to inhibit the autocleavage of the main protease.

## Significance

The coronavirus main protease (M^pro^) is required for viral replication. Here, we obtained the extended conformation of the native monomer of severe acute respiratory syndrome coronavirus 2 (SARS-CoV-2) M^pro^ by trapping it with nanobodies and found that the catalytic domain and the helix domain dissociate, revealing allosteric targets. Another monomeric state is termed compact conformation and is similar to one protomer of the dimeric form. We designed a Nanoluc Binary Techonology (NanoBiT)-based high-throughput allosteric inhibitor assay based on structural conformational change. Our results provide insight into the maturation, dimerization, and catalysis of the coronavirus M^pro^ and pave a way to develop an anticoronaviral drug through targeting the maturation process to inhibit the autocleavage of M^pro^.

The current COVID-19 pandemic caused by severe acute respiratory syndrome coronavirus 2 (SARS-CoV-2) is posing a global health crisis, and the evolving variants of SARS-CoV-2 have reduced the efficacy of the vaccine (1–3). In addition, in less than 20 y, there have been two other coronavirus epidemics: SARS-CoV and MERS-CoV (4). There is thus an urgent need to develop antiviral drugs against a variety of coronaviruses. The viral main protease, known as M^pro^, or 3CL^pro^ (5), plays an essential role in the polyprotein processing of viral replication. Therefore, M^pro^ is an attractive target for the design and development of antiviral drugs (6–8).

The coronavirus M^pro^ has been widely explored through structural and biochemical research (5, 7–10). The crystal structure of coronavirus M^pro^ presents as a symmetric homodimer. Each monomer comprises a catalytic domain (β-barrel fold, including domains I and II) and an α-helical domain (domain III), where the two domains are connected by a long loop region. The catalytic dyad (H41 and C145) is located in the cleft between domain I and domain II of the β-barrel fold. Dimerization has been found to be necessary for the enzyme to maintain an enzymatically active conformation. However, there is an equilibrium between the inactive monomer and active dimer, and the activity of the enzyme is limited by its concentration and the dissociation constant (5–7, 9, 10).

Most studies have focused on the active dimeric M^pro^, with the development of antiviral drugs, such as peptidomimetic and nonpeptidic compounds that act directly against the active site and that are based on the structure of the active dimer (5, 10–12). Targeting the folding process may be an effective alternative strategy to suppress the autocleavage of M^pro^ in the viral polyprotein. In order to inhibit the maturation of M^pro^, analysis of the conformation of the monomeric state is required. However, it is difficult to obtain the structure of authentic monomeric M^pro^ because it is subject to forming a dimer at high concentrations by crystallization. The camelid single-domain antibodies (nanobodies) are able to stabilize the intermediate states of the dynamic protein through inserting a relatively long complementarity determining region 3 (CDR3) into the clefts of the antigen. This enabled them to become widely used tools to capture the specific conformational states of flexible proteins to understand their mechanism and regulate protein activity (13–15). We utilized these nanobodies to trap the monomeric specific conformation of SARS-CoV-2 M^pro^.

Conformational change reveals the allosteric target, and based on the structural conformational change, a high-throughput activity assay can be designed to screen leading allosteric inhibitors. Nluc has been widely studied for the development of a cleavage luciferase reporter system, Nanoluc Binary Technology (NanoBiT) (16). The luminescent signal from NanoBiT provides an accurate indication of the interaction dynamics. The NanoBiT system has been successfully used to detect protein–protein interactions and to design biosensors based on the conformational changes, establishing high-throughput screening approaches to identify allosteric modulators (17, 18).

Here, we first identified a set of allosteric inhibitory nanobodies against M^pro^, and then determined two authentic monomeric transient structures of SARS-CoV-2 M^pro^ trapped by two nanobodies (NB1A2 and NB2B4). One is the extended state stabilized by NB2B4, in which the catalytic domain and extra helix domain is dissociated, and reveals the allosteric target. It is the precursor of all other states. The other conformation is similar to that of the dimer, and displays a substantial compaction compared with the extended structure, and is thus termed the compact conformation. Both nanobodies can target dimerization of M^pro^ and dissociate the dimer into monomers, thereby exerting an inhibitory effect on M^pro^; thus, they are allosteric inhibitors. The epitope of NB1A2 on M^pro^ overlaps with the catalytic and substrate binding sites, so it is also a competitive inhibitor. In addition, we established an innovative NanoBiT-based high-throughput allosteric inhibitor assay based on the conformational changes of monomeric M^pro^, and this kind of allosteric modulator can inhibit autocleavage.

## Results

### Generation and Characterization of Camelid Inhibitory Nanobodies against SARS-CoV-2 M^pro^.

**Fig. 1.**
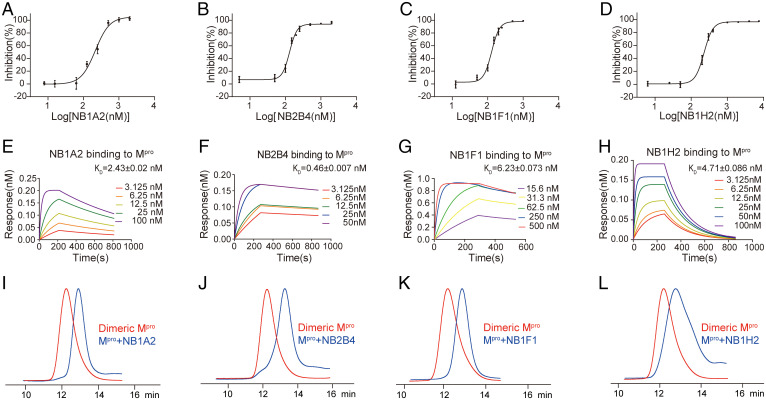
Generation and characterization of camelid inhibitory nanobodies against SARS-CoV-2 M^pro^. (*A–D*) The hydrolytic activity of SARS-CoV-2 M^pro^ was measured in the presence of increasing concentrations of the inhibitory nanobodies NB1A2 (*A*), NB2B4 (*B*), NB1F1 (*C*), and NB1H2 (*D*). (*E–H*) Biolayer interferometry binding kinetics measurements for NB1A2 (*E*), NB2B4 (*F*), NB1F1 (*G*), and NB1H2 (*H*) (*K*_D_, equilibrium dissociation constant). (*I–L*) Representation of SEC profiling of the dimeric M^pro^ (red) and dimeric M^pro^ + NB complex (blue); dimeric M^pro^ + NB1A2 complex (*I*), dimeric M^pro^ + NB2B4 complex (*J*), dimeric M^pro^ + NB1F1 complex (*K*), and dimeric M^pro^ + NB1H2 complex (*L*).

We developed nanobodies with potent inhibitory activity against the SARS-CoV-2 main protease. We first obtained 11 high-affinity nanobodies from the M^pro^-immunized camel using the phage display library (Table 1), and identified seven nanobodies with a potent inhibitory effect on the catalytic activity of M^pro^; the IC_50_ values ranged from 102.043 ± 16.935 nM to 186.633 ± 23.081 nM (Fig. 1 *A–D* and *SI Appendix*, Fig. S1 *A–D*). The most potent nanobodies, NB1A2 and NB2B4, were 186.633 ± 23.081 nM and 122.000 ± 7.711 nM, respectively. We measured their binding kinetics using biolayer interferometry, and NB1A2 and NB2B4 had binding affinities of 2.426 ± 0.020 nM and 0.461 ± 0.007 nM, respectively (Fig. 1 *E–H* and *SI Appendix*, Fig. S1 *E–K*). Interestingly, the profiles from size-exclusion chromatography (SEC) showed that the result of these inhibitory nanobodies binding to the dimeric M^pro^ was dissociation to catalytically inactive monomers (Fig. 1 *I–L*). Moreover, NB1A2 and NB2B4 have different epitopes on M^pro^ because they can simultaneously bind to M^pro^, which indicates that both nanobodies are allosteric inhibitors, which can convert the active dimeric M^pro^ into the inactive monomeric M^pro^, and each has a different allosteric inhibitory mechanism for SARS-CoV-2 M^pro^ (*SI Appendix*, Fig. S1*L*).

**Table 1.** The CDR sequences of the nanobodies

	CDR1	CDR2	CDR3
NB1A2	GVTASSVY	INTVGYT	AATYLLRFASLSATNFPY
NB2B4	GYTYSSKC	IYTGGGST	AASGAIAGIRLCLPGHTFYTY
NB1H2	GATASSVL	INTVGYT	AATYLLRFAPLSATDFPY
NB1F1	RYTFSVGC	IYPGGGST	AAPSAASTCRSLRLGMNGVFSY
NB1A1	GFTSSDYN	ITSTMRT	ATDSSGDQ
NB1D5	GYTASRYC	IDSNGRT	AAHDLLYGGVLRCGVARY
NB1D10	GYSYCNYD	IDSDGNT	KVGSIASSVPEVSCPPSAPFGY
NB1B12	GYSYCNYD	IDSDGNT	KVGSIASSVPERSCPPSAPFGY
NB2D10	GHTYSYYC	IDSDDST	AATHYTRRGLLFRLLADFGY
NB2E3	GFIATSCA	ITTDGTT	KLCCSGQYCA
NB2H4	GVAVSSVL	INAVGYT	AATYLLRTASLSASNFPY

### Two Transient States of Monomeric M^pro^ Are Trapped by Two Different Allosteric Inhibitory Nanobodies.

**Fig. 2.**
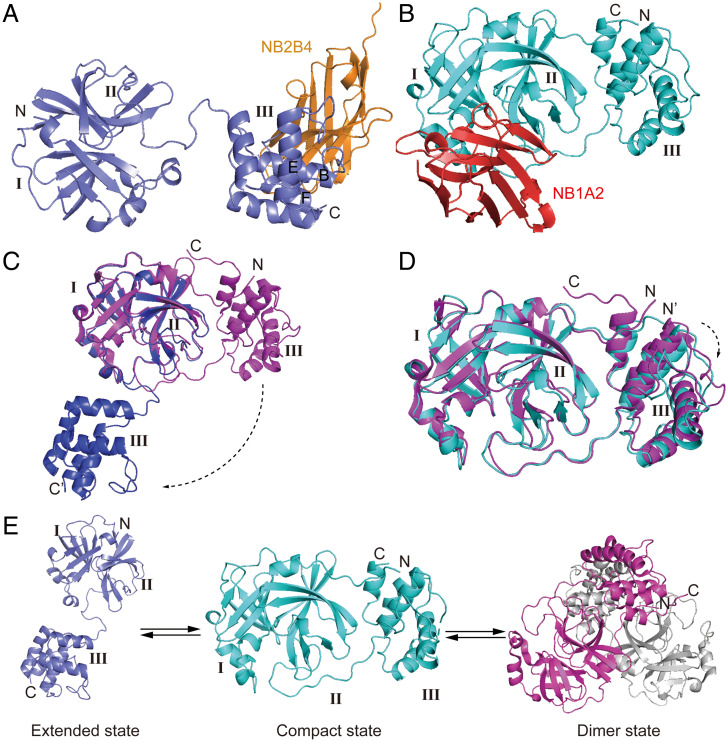
Two transient conformational of SARS-CoV-2 monomeric M^pro^ are captured by NB2B4 and NB1A2. (*A*) Crystal structure of SARS-CoV-2 monomeric M^pro^ in complex with NB2B4, with the monomeric M^pro^ colored in blue and NB2B4 colored in orange, respectively. SARS-CoV-2 monomeric M^pro^ presents an extended conformation. (*B*) Crystal structure of SARS-CoV-2 monomeric M^pro^ in complex with NB1A2, with the monomeric M^pro^ colored in cyan and NB1A2 colored in red, respectively. SARS-CoV-2 monomeric M^pro^ shows a compact conformation. (*C*) Superposition of the extended M^pro^ (blue) and active protomer of dimeric M^pro^ (magenta, PDB ID code 7LMC) based on the catalytic domain. (*D*) Superposition of the compact M^pro^ (cyan) and active protomer of dimeric M^pro^ (magenta, PDB ID code 7LMC). (*E*) The equilibrium among the extended monomers, the compact monomer, and the dimer of SARS-CoV-2 M^pro^.

To obtain the structure of authentic monomeric M^pro^, we selected two nonoverlapping nanobodies (NB2B4 and NB1A2) and then cocrystalized M^pro^ in complex with NB2B4 and NB1A2, respectively. We determined two complex structures at a resolution of 2.0 Å and 1.75 Å by molecular replacement (Fig. 2 *A* and *B* and Table 2).

**Table 2.** Data collection and refinement statistics

	M^pro^_NB2B4	M^pro^_NB1A2
Wavelength	0.979	0.979
Resolution range	33.21–2.0 (2.03–2.0)	29.88–1.75 (1.78–1.75)
Space group	P 21 21 2	P 43 21 2
Unit cell	254.6 33.5 48.7 90 90 90	61.7 61.7 239.0 90 90 90
Unique reflections	29,088 (1,394)	48,189 (2,351)
Multiplicity	12.1 (10.8)	23.3 (19.0)
Completeness (%)	98.6 (95.9)	100 (100)
Mean I/σ(I)	26.73 (3.28)	20.3 (2.25)
Wilson B-factor(Å^2^)	23.44	18.50
*R*_merge_	0.089(0.719)	0.292 (2.378)
*R*_meas_	0.093 (0.755)	0.298 (2.442)
*R*_pim_	0.026(0.222)	0.060 (0.550)
CC1/2	0.997 (0.846)	0.999 (0.930)
Reflections used in refinement	28,360	47,780
Reflections used for *R*_free_	1,421	2,305
*R*_work_	0.23	0.19
*R*_free_	0.26	0.22
No. of nonhydrogen atoms	3,449	3,700
RMS (bonds) (Å)	0.004	0.004
RMS (angles) (°)	0.70	0.71
Ramachandran favored (%)	96.59	98.55
Ramachandran allowed (%)	3.41	1.45
Ramachandran outliers (%)	0.00	0.00

The structure of M^pro^ in complex with NB2B4 shows that it binds to the α-helical domain of M^pro^, and is far from the catalytic site and substrate binding sites (Fig. 2*A*). NB2B4 binding causes the separation of the α-helical domain and antiparallel-barrel fold, the hairpin loop linking domains II and III fully unfolds, the α-helical domain swings 100° from one side of the β-barrel structure to the other side, and there is a change from bound state to free state (Fig. 2*C*). The NB2B4 binding did not result in overall conformational change to either the α-helical and β-barrel catalytic domains as two independent fold units, compared with that of dimeric M^pro^. The conformation of monomeric M^pro^ is designated as the extended state.

The crystal structure of M^pro^ in complex with NB1A2 shows that the epitope of NB1A2 overlaps with the substrate binding site and catalytic site (Fig. 2*D*). The catalytic domain and helix domain contact each other, representing the compact conformation of monomeric M^pro^ compared with the above extended conformation, and the whole architecture is similar to that of one promoter of dimeric M^pro^. However, the α-helical domain displaces from the catalytic domain about 2 Å (Fig. 2*D*). The structure also differs from the previously reported structures of monomeric mutants in which the catalytic domain and helix domain rotated about 40° compared with the compact monomer and the dimeric M^pro^ (19–22) (*SI Appendix*, Fig. S2*A*). The monomeric structure shows that the interface between the C-terminal and N-terminal domains is mainly composed of loop–loop interactions, including the salt bridge formed by R131 with D197, D289, and E290, and a hydrogen bond between N133 and D197 (*SI Appendix*, Fig. S2*B*), which indicate the C-terminal and N-terminal domains are easily dissociated. In addition to the two distinct conformations of SARS-CoV-2 monomeric M^pro^ stabilized by two nonoverlapping nanobodies, the mutant monomeric structures of SARS-CoV M^pro^ were previously reported (19–22). They adopted three completely different conformations, which indicates that M^pro^ in the inactive monomer has conformational heterogeneity (*SI Appendix*, Fig. S2*A*). Our data support that the compact state may be an intermediate and the monomeric extended state may be the parent state of two other monomeric forms. The two folding units of extended monomeric state come together to form the compact monomeric states, and then dimerize into the active dimer (Fig. 2*E*).

### Mechanism of Allosteric Inhibition of SARS-CoV-2 M^pro^ by NB2B4.

**Fig. 3.**
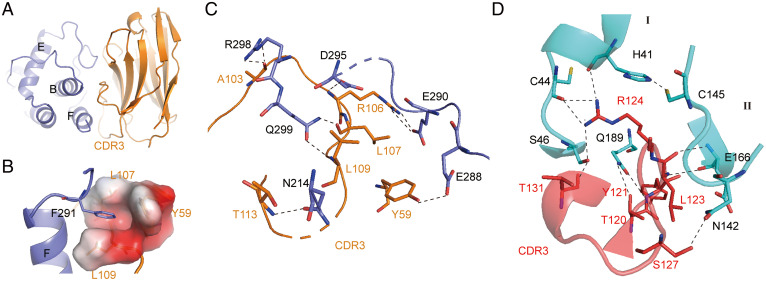
Structural mechanism of inhibition of SARS-CoV-2 M^pro^ by NB2B4 and NB1A2. (*A*) The epitope of NB2B4 on the C-terminal domain of SARS-CoV-2 M^pro^, with the C-terminal domain colored in blue and NB2B4 colored in orange, respectively. (*B*) The residues F291 of the F helix from the C-terminal domain are inserted into the hydrophobic pocket composed of residues Y59, L107, and L09 of NB2B4. Residue F291 is shown as a stick model; residues Y59, L107, and L09 of NB2B4 are shown as the electrostatic surface to illustrate the hydrophobic pocket. (*C*) Close-up views of the interface between SARS-CoV-2 extended M^pro^ (blue) and NB2B4 (orange). (*D*) Close-up views of the interface between SARS-CoV-2 compact M^pro^ (cyan) and NB1A2 (purple). The key residues involved in interaction are shown as stick models. Polar interactions are indicated with black dashed lines.

The epitope of NB2B4 on the C-terminal domain of the monomeric M^pro^ consists of the F helix and the loop connecting the E and F helices (Figs. 2*A* and 3*A*). The aromatic ring of residues F291 from the loop between the E and F helices inserts into the hydrophobic pocket composed of L107, L109, P110, and Y59 of NB2B4 (Fig. 3*B*). Mutation of F291 to Ala destroys the hydrophobic interaction, thereby eliminating the binding of NB2B4 to M^pro^ (*SI Appendix*, Fig. S3*A*). At the periphery of the hydrophobic core, there are multiple hydrophilic interactions between NB2B4 and M^pro^ (Fig. 3*C*). First, R106 of CDR3 forms a salt bridge with the carbonyl group of sidechain of E290 from the loop between the E and F helices, and donates the hydrogen bond with the sidechain carbonyl group of helix F D295 (Fig. 3*C* and *SI Appendix*, Fig. S2*B*). Second, the mainchains of L107 and L109 from CDR3 form double hydrogen bonds with the sidechain of Q299 from helix F. Third, an electrostatic interaction between the sidechain of R298 and the mainchain of A103 of NB2B4 is observed (Fig. 3*C*). The sidechain–sidechain interaction is between T113 from NB2B4 and N214 of the C-terminal domain (Fig. 3*C*). In addition, the hydroxyl group of Y59 on NB2B4 also forms one hydrogen bond with E288 from the C-terminal domain. The binding of NB2B4 to M^pro^ disrupts the important elements of enzyme dimerization, and also destroys the interactions between the N-terminal and C-terminal domains. Therefore, NB2B4 can stabilize the monomeric extended conformation and prevent the formation of the monomer compact state and dimer state.

It was previously reported that N-finger was crucial for the dimerization and enzyme activity (19, 23, 24). The density of N-terminal residues (residues 1–11) in the extended monomeric M^pro^ is completely missing compare with the active dimeric M^pro^, which is due to the loss of contact with its own C-terminal domain and the interaction from other protomers. We superimposed the C-terminal domain of the extended state of monomer M^pro^ and the corresponding part of one protomer in the active dimeric M^pro^, and found that the NB2B4 has steric clash with the whole structure of another protomer. It was also reported that the unstructured loop consisting of residues 284 to 286 of the helical domain III of each protomer form a complementary interface, which contributes to the stabilization of SARS-CoV-2 M^pro^ (25); however, the binding of NB2B4 to the helical domain III disrupts the association between two protomers. NB2B4 also interferes with the interaction between domain III and domain II (*SI Appendix*, Fig. S3 *B* and *C*). This implies that NB2B4 blocks the contact between the N-terminal and C-terminal domains and inhibits enzyme dimerization. Therefore, NB2B4 is specific to the extended monomeric M^pro^, and locks the monomeric M^pro^ in the extended conformation.

### NB1A2 Is Not Only an Allosteric Inhibitor, but Also a Competitive Inhibitor.

The structure reveals that NB1A2 employs its CDR3, inserting into the cleft of the substrate binding site and the catalytic site as a competitive inhibitor (Fig. 2*B*). This creates extensive intimate interactions through hydrogen bond networks (Fig. 3*D*). The sidechain of R124 on CDR3 forms hydrophilic interactions with the catalytic site residue H41 and the two residues C44 and S46 from the substrate binding pocket. A hydrogen bond is formed between the carboxy group of T131 on CDR3 and the sidechain hydroxy group of S46 from helix A. Q189 from the loop connecting domains II and III interacts with residues T120, Y121, and L122 of CDR3 through four different hydrogen bonds. These interactions can block the substrate binding. The sidechain of N142 donates a hydrogen bond to the sidechain of S127 of CDR3. In addition, a hydrogen bond is formed between the mainchain of E166 and the mainchain of L123 from NB1A2. However, the two residues, N142 and E166, are crucial to form the oxyanion hole and the subsite S1 pocket. Moreover, E166 also plays an important role in the enzyme dimerization (25, 26).

The N-terminal finger in the compact monomer shows that it swings about 21.5° relative to that of active dimer and the orientation of the sidechain of M6 and R4 changes greatly (*SI Appendix*, Fig. S4*A*). It is possible that the N-terminal finger of the monomer is not restrained by the interaction with the other protomer of dimeric form. The architecture of monomeric M^pro^ supports the view that the N-finger plays a key role for the maturation, dimerization, and enzyme activity (19, 23, 24).

In dimeric M^pro^ structure, three hydrogen bonds are formed among the C-terminal residues Q299, V303, and F305 from the A protomer and the residues P122, S123, and S139 from the B protomer (5–7, 9, 10). Thus, the orientation of the A protomer C terminus is stabilized by the B protomer. Then, three hydrogen bonds are formed between the residues Q306 from the C terminal and the residues K12, Y154, and D155 from the catalytic domain, which fixes the distance between the C-terminal and N-terminal domains (5–7, 9, 10) (*SI Appendix*, Fig. S4 *B* and *C*). However, these interactions are lost in the monomer, and the constraints to the C terminal are released; indeed, the density of the residues of the C terminal is not observed in the monomer. Therefore, the orientation and distance between the α-helical domain and the catalytic domain is related with M^pro^ dimerization. The two domains present a different rearrangement in the monomer because the C-terminal residues are not restricted, and there are at least two different structures: our compact structure and the monomeric mutant structure, which indicates that the C-terminal residues play a vital role for the dimerization and catalytic activity.

The superposition of the NB1A2 binding monomer and one protomer of dimeric M^pro^ shows that the NB1A2 has a steric clash with the B helix, D helix, and C terminal of another protomer of dimeric M^pro^ (*SI Appendix*, Fig. S4*D*). This is one of the reasons why NB1A2 can target dimerization of M^pro^. Therefore, NB1A2 also acts as an allosteric inhibitor of the enzyme.

### The Conformation of Monomeric M^pro^ Is Incompatible with Substrate Binding.

**Fig. 4.**
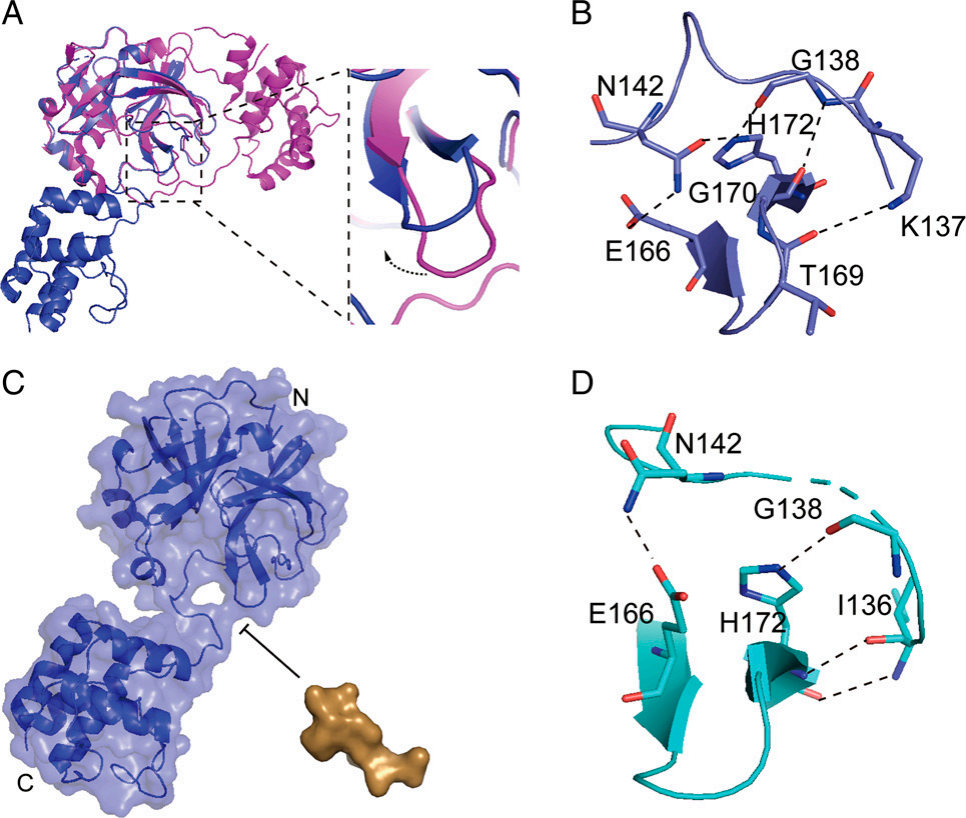
The conformation of monomeric M^pro^ is not suitable for the substrate binding. (*A*) Superposition of the extended monomeric structure and one protomer of dimeric structure based on the N-terminal domain, with the extended structure colored in blue, and dimer structure colored in magenta (*Left*) and close-up view of the conformational change of the β-turn (*Right*). (*B*) The interaction between the β-turn (E166-H172) and the active loop (K137-N142) of the extended monomeric structure (blue). (*C*) The model of the extended monomer M^pro^ and substrate (TSAVLQ, derived from the N-terminal autocleavage sequence of the viral protease), the volume of substrate bound pocket is insufficient to accommodate the substrate. The extended monomer M^pro^ and substrate are shown as surface, with the monomeric M^pro^ colored in blue and substrate colored in brown. (*D*) The interaction between the β-turn (E166-H172) and the active loop (K136-N142) of the compact monomeric structure (cyan). The key residues involved in interaction are shown as stick models. Polar interactions are indicated with black dashed lines.

Compared with the active dimer, the extended monomer displays an upward rotation of the β-turn composed of residues 166 to 172 (Fig. 4*A*), and the β-turn approach the active loop, consisting of residues 145 to 137, because E166, H172, G170, and T169 from the β-turn form hydrogen bond networks with N142, G138, and K137 from the active loop, resulting in the closure of the S1 subsite (Fig. 4*B*). The β-turn also shifts to the left and the volume of the S2-S4 binding pocket shrinks down, which is not suitable for the substrate binding (Fig. 4*C*). The amplitude of upward movement of the β-turn in the compact monomer is a little smaller than that of the extended monomer because the pattern of the hydrogen bonds pattern has slightly changed: some hydrogen bonds are untied (such as the hydrogen bond between the sidechain of N142 and H172, the hydrogen bond between the mainchain of G138 and G170, and the hydrogen bond formed by K137 and T169), which releases some constraints between the β-turn and active loop, and provides more space for the P1 binding. However, the S1 subsite is still closed due to the existence of hydrogen bond formed by N142 and E166 (Fig. 4*D* and *SI Appendix*, Fig. S5*A*). Alignment of the β-turn in different states shows that it gradually displaces, rendering more and more space for substrate binding, from the extended monomer to the compact monomer, then to the inactive protomer of the dimer, and finally to the active protomer of the dimer (*SI Appendix*, Fig. S5 *B–G*). The volume of substrate bound pocket in all different states except for the active protomer is insufficient to accommodate the substrate; therefore, the substrate binding pocket is opened step by step with the maturation of the enzyme.

The orientation of the active loop composed of residues 137 to 145 is crucial for enzyme activity (5–7, 9, 10). All residues in the active loop of the monomer have large structural dislocation, compared with the active loop conformation of dimeric M^pro^. In the active dimer of M^pro^, the aromatic ring stacking for Phe140 and His163 and the two hydrogen bonds between the carboxylate groups of Glu166 and Phe140 and the NH-group of Ser1 from another protomer stabilize the right conformation of the oxyanion loop and S1 pocket of the substrate-binding site (25). But, in the monomeric M^pro^, the interactions between two rings of F140 and Y126 replace the aromatic ring stacking for Phe140 and His163, and the N-terminal finger displaces (*SI Appendix*, Fig. S6) (25). As a result, the active loop consisting of residues 137 to 145 shifts to the right and has significant conformational changes, resulting in the collapses of the oxyanion hole and the closure of the S1 pocket (*SI Appendix*, Fig. S6). The whole conformation of the loop in the monomeric M^pro^ is very similar to that of the inactive protomer of the dimer and the monomeric mutants (13, 14, 21, 22). Therefore, the active site in the monomeric M^pro^ does not adopt the right conformation for the substrate binding.

### Development of a NanoBiT-Based Conformational Sensor for the Monomeric SARS-CoV-2 M^pro^.

**Fig. 5.**
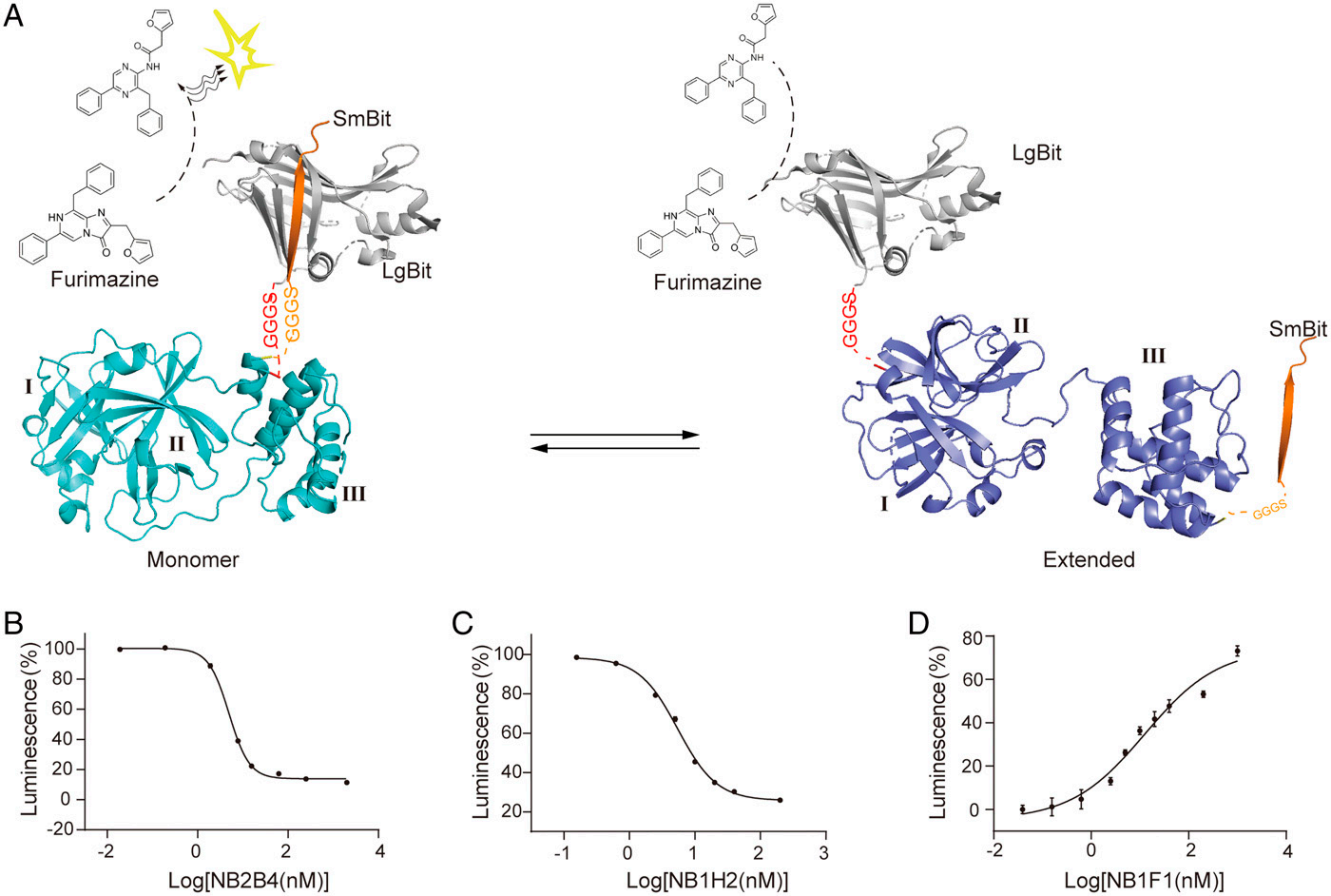
Establishing a NanoBit-based conformational sensor for the monomeric SARS-CoV-2 M^pro^. (*A*) Schematic of NanoBiT complementation to examine conformation changes in monomeric M^pro^. Crystal structures of the compact state colored in cyan (*Left*) and the extended conformation colored in blue (*Right*). The M^pro^ was fused at its N terminus with LgBiT (gray) and at C terminus with SmBiT (yellow). The GGGS linker was inserted between N terminus/C terminus and LgBiT/SmBiT. The N-terminal LgBiT was expected to bind in close proximity to the C-terminal SmBiT in the compact conformation of M^pro^, and increase the possibility of LgBiT-SmBiT complementation and the complemented luminescence, while it decreases in the extended conformation. (*B–D*) Luminescence signal measured for NB2B4 (*B*), NB1H2 (*C*), and NB1F1(*D*) in different concentrations; the pIC_50_ values for NB2B4 and NB1H2 are 4.94 nM and 5.48 nM, respectively; the pEC_50_ of NB1F1 is 12.61 nM. Data are mean ± SEM of at least three independent experiments performed in triplicates and normalized to the maximum response of the control.

As revealed in the structural analysis, there is significant conformational rearrangement between the extended and compact monomeric M^pro^. The interaction between the catalytic domain and helix domain is accompanied by the proximity of the N terminal and C terminal of monomeric M^pro^. The LgBiT (large subunit) and SmBiT (small subunit) fused to the N terminal and C terminal of monomeric M^pro^, respectively (Fig. 5*A*). Complement luminescence is used to specifically monitor the association of the catalytic domain and helix domain. Because the residues extension in native sequence to the N and C termini significantly increase the dimerization *K*_d_ compared with the mature M^pro^ (27), the profile from SEC showed that the purified NanoBiT-M^pro^ (LgBiT-M^pro^-SmBiT) is a monomer (*SI Appendix*, Fig. S7). As expected, the results showed that the luminescent signal was strong in the absence of NB2B4 and the reducing luminescence was observed in the presence of NB2B4, which clarifies the conformational changes associated with the action of NB2B4 (Fig. 5*A* and *SI Appendix*, Fig. S8*A*). The inhibitory effect of NB2B4 on the M^pro^ was determined by the NanoBiT sensor assay, with a pIC_50_ of 4.94 nM (Fig. 5*B*). We also tested other strongly inhibitory nanobodies we identified. The results showed that in the presence of NB2B4, NB1H2, or NB1A1, the luminescence decreases, while in the presence of NB2H4, NB1D5, NB1A2, or NB1F1, the luminescence signal increases and the effects are different (*SI Appendix*, Fig. S8*A*). This confirmed that there was equilibrium between the M^pro^ extended conformation and compact conformation in solution. Therefore, in solution, it is possible that there is an equilibrium among the extended state, the compact state, and the active dimer.

The preliminary test showed that in addition to NB2B4, NB1H2 also has a strong inhibitory effect on the luminescence signal. So, we further tested that the inhibitory effective curve of NB1H2 and its pIC_50_ value was 5.48 nM (Fig. 5*C*). We also tested the dose-dependent enhancement curves of NB1F1, NB2H4, NB1A2, and NB1D5 that have effect of increasing the luminescent signal with the pEC_50_ about 12.61 nM, 15.68 μM, 15.76 nM, and 41.26 nM (Fig. 5*D* and *SI Appendix*, Fig. S8 *B–D*).

The interaction between the catalytic domain and helix domain is necessary for the compact state of monomeric M^pro^, which is a prerequisite state for the active dimeric M^pro^. Therefore, the allosteric modulators blocking the interaction of the two domains could be therapeutically valuable. We developed the innovative NanoBiT-based high-throughput allosteric inhibitor assay based on the conformational transition between the monomeric M^pro^ extended state and the monomeric M^pro^ compact state, not based on the conformational rearrangement between the monomeric M^pro^ compact state and the active dimer. Therefore, the variation of luminescence signal indicates the conformation transition between the monomeric M^pro^ extended state and the monomeric M^pro^ compact state. The decrease of luminescence signal means that the inhibitor stabilizes the monomeric M^pro^ extended conformation, while the increase of luminescence signal indicates that the inhibitor stabilizes the monomeric M^pro^ compact conformation. The high-throughput screening method is feasible for screening allosteric inhibitory nanobody or compounds through targeting the folding process to inhibit the activity of dimeric M^pro^ and also prevent the M^pro^ in the polyprotein assembling into any active form and inhibit the self-cleavage.

This assay was used to measure the interaction between the two domains of M^pro^. According to the established assay, we can use the purified LgBiT-M^pro^-SmBiT protein to screen allosteric inhibitors, and it is further possible to use the transfected cell with the plasmids encoding LgBiT-M^pro^-SmBiT to screen allosteric inhibitors under different experimental conditions in the microplate format. The assay is helpful for the high-throughput screening of M^pro^ allosteric inhibitors.

## Conclusion

In this study, we identify a set of allosteric inhibitory nanobodies against M^pro^, and determined the two crystal structures of M^pro^ with two noncompeting nanobodies to clarify their inhibitory mechanism. The two nanobodies captured two distinct transient conformations of M^pro^ in the inactive monomeric state. One monomer has an extended conformation with its N-terminal and C-terminal domain dissociated, and the other monomer has the same structure as that of the protomer of the well-characterized dimer. Complemented with previously reported numbers of dimeric structure (5–7, 9, 10, 28, 29), these results allow us to understand the structural framework and essential events during the maturation of M^pro^ (Fig. 2*E*). The extended state and compact state should exist in an equilibrium in the polyprotein, and based on the structural rearrangement, the screening inhibitor can prevent M^pro^ autocleavage.

The maintained dissociation of the N- and C-terminal domains is an early stage interfering with enzyme maturation, which can prevent the M^pro^ in the polyprotein assembling into any active form and inhibit the self-cleavage. Therefore, targeting early maturation of the main protease is an effective strategy for the development of antiviral drugs. Based on the structural rearrangement, an innovative NanoBiT-based high-throughput allosteric inhibitor assay was developed to suppress the autocleavage of M^pro^ in the viral polyprotein. In addition, the allosteric nanobodies unveil novel promising allosteric targets for the structure-based design of allosterically small molecular inhibitors.

## Materials and Methods

### Cloning, Expression, and Purification of SARS-CoV-2 M^pro^.

The cDNA of full-length SARS-CoV-2 M^pro^ was code-optimized and inserted into pGEX4T-1 vector by Genscript, which encoded the SARS-CoV-2 M^pro^ protease (accession no.: MN908947.3, ORF1ab polyprotein residues 3264 to 3569), and the LVPRGS was inserted into the N terminus of the M^pro^ as the linking sequence between the GST and M^pro^, which is the specific substrate recognition sequence of thrombin. After transformation into BL21(DE3), cells were cultured in the 2YT medium supplement with ampicillin (50 µg/mL) and induced with isopropyl β-d-1-thiogalactopyranoside (IPTG, final concentration 0.1 mM) at 16 °C for 10 h, when the value of OD_600_ reached 0.6. Then, the cells were sedimented by centrifuge and resuspended in the lysis buffer (1×PBS) with protease inhibitor, and lysed by sonication. Next, they were centrifuged at 10,000 rpm for 45 min, the supernatant was loaded into the equilibrated GST-column and washed with PBS for 30 mL, and the GST-tagged M^pro^ was eluted by adding reduced glutathione to the elution buffer. The elution was concentrated and the GST-tag was cleaved off using a thrombin protease (Yeasen, Cat#:20402ES03). Finally, it was purified by gel-filtration chromatography using a HiLoad 16/600 Superdex 75 pg column (Cytiva, Cat#:28989333) and stored into liquid nitrogen.

To obtain homodimeric M^pro^, the construction method of expression vector was as described previously (10, 30). The gene encoding M^pro^ was cloned into the pGEX6p-1 vector, and AVLQSGFR was inserted into the N terminus of the M^pro^ gene as the linking sequence between the GST gene and M^pro^ gene, which is the specific substrate recognition sequence of SARS-CoV2 M^pro^; the authentic N terminus would become available by autocleavage during protein expression. Additionally, the GPHHHHHH were inserted into C terminus, which can be cleaved by the rhinovirus 3C protease to generate the native C terminus of M^pro^. The expression purification strategy was as described above.

### Construction of Camelid Nanobody Phage-Display Library.

Camel immunizations and nanobody library generation were performed as described previously (31). Animal work was conducted under the supervision of Shanghai Institute of Materia Medica, Chinese Academy of Sciences. Two camels were immunized with the purified M^pro^ at doses of 1 mg once a week seven times. After immunization, the peripheral blood lymphocytes were isolated from the whole blood using Ficoll-Paque Plus according to the manufacturer’s instructions. Total RNA from the peripheral blood lymphocytes was extracted and reverse-transcribed into cDNA using a Super-Script III FIRST-Strand SUPERMIX Kit (Invitrogen). The VHH encoding sequences were amplified with two-step enriched-nested PCR using VHH-specific primers and cloned into pMECS vector and transformed into electro-competent *Escherichia coli* TG1 cells (Lucigen). The size of the constructed nanobody library was evaluated by counting the number of bacterial colonies. Colonies were harvested and stored at −80 °C.

### Nanobody Library Screening.

To screen and identify the specific nanobody of M^pro^, we used the phage-display technology and bio-panning for two rounds. For each round of bio-panning, the biotinylated M^pro^ was combined at the bottom of the 96-well microtiter plates (ThermoFisher), Then, the phages and expressed nanobody were dispensed and incubated for 2 h, and washed with PBST 10 times and with PBS 5 times. The phages were collected and infected TG1 cells. The positive clones were selected by M^pro^-coated ELISA plates for sequencing.

### Expression and Purification of Nanobodies.

The specific nanobody of M^pro^ was cloned into pMECS vector and transformed into Top10F′ cells (Huayueyang Biotech) to inoculated in 2YT medium containing 100 μg/mL ampicillin, 0.1% (wt/vol) glucose, and 1 mM MgCl_2_. When the OD_600_ reached 0.6 to 0.8, the IPTG was added, and when the work concentration was 1 mM, after 16 h, we harvested the cells and resuspended them in the lysis buffer and they were lysed by sonication. The supernatant was load onto Ni-NTA beads and eluted with elution buffer, which contained 20 mM Hepes pH 7.5, 150 mM NaCl, and 300 mM imidazole. The elution was concentrated by the 3-kDa concentrated tube of Amicon Ultra Filter and polished by purified by gel-filtration chromatography using a HiLoad 16/600 Superdex 75-pg column (Cytiva, Cat#:28989333) and stocked into liquid nitrogen.

### Analysis of Nanobody Binding Epitope on M^pro^.

In order to analyze the nanobody binding epitope on M^pro^, the purified dermic M^pro^ and purified nanobodies were mixed and incubated on ice for 30 min. The SEC experiment was performed and the peak position of the mixture was detected by HPLC (Agilent 1260) with the Superdex 200 increase 5/150 GL (Cytiva).

### Crystallization and Data Collection.

The purified M^pro^ and NB2B4 or NB1A2 were incubated at 4 °C for 30 min, then polished by the HiLoad 16/600 Superdex 75-pg column, and the peak collected for crystallization. The complex of the M^pro^ and NB2B4 was crystalized using the sitting-drop vapor-diffusion method at 20 °C for 2 wk. The crystals were obtained in conditions containing 9% PEG 20,000, 0.1 M MES (pH5.5). The crystals of the M^pro^ in complex with NB1A2 were grown in 0.2 M (NH_4_)_2_HPO_4_, 20% PEG3350, pH 8.0. X-ray diffraction data were collected at 100K on the beamline 19U1 at the National Synchrotron Radiation Research Center, China (λ = 0.978 Å) (32). Data were processed using HKL3000 suite.

### Structure Determination.

The structure determination of the complex of the M^pro^ and NB2B4 or NB1A2 was solved with the molecular replacement using Phaser in the PHENIX package (33), and the model (PDB ID code 7LMC) of SARS-CoV-2 M^pro^ was used. Model building was carried out by Coot (34). The refinement of the structure was done with PHENIX suite. Finally, stereochemical quality and final validation of the model were performed using Mol Probity (35). The final statistics of data collection and structural refinement are shown in Table 2. Figures were prepared in PyMOL (https://pymol.org/2/).

### Enzymatic Activity and Inhibition Assay.

The activity of SARS-CoV-2 M^pro^ was measured by a continuous kinetic assay with the substrate MCA-peptide (MCA-AVLQSGFR-Lys [Dnp]-Lys-NH2), a peptide similar to the N-terminal or C-terminal amino acid sequence of the nsp5 to nsp16 of SARS-CoV-2, which was synthesized by GL Biochem Ltd. Inhibition kinetics were performed by incubating 200 nM enzyme with 20 nM to 5 μM inhibitor in assay buffer at 4 °C with gentle shaking for 30 min, and then adding into the black half-area 96-well plate, 50 μL of 40 μM substrate into the well to initiate reactions. The fluorescence signal changes of MCA were detected at the excitation wavelength 320 nm and emission wavelength 405 nm. The results were compared with the control sample without inhibitor, and the nanobodies with certain inhibitory effect were screened out. The initial rate of the reaction was obtained, and then the IC_50_ was calculated by GraphPad Prism7 software.

Under experimental conditions, the inhibition percentage of the reaction was calculated compared with the activity of M^pro^ in the absence of nanobodies. The formula for calculating IC_50_ is *v* = *a*/(1 + ([*I*]/IC_50_). In the formula, *v* is the initial reaction rate of the reaction in the presence of nanobodies, *a* is the enzymatic reaction rate in the absence of nanobodies, [*I*] is the working concentration of nanobodies, and IC_50_ is the concentration of nanobodies when the activity of the enzyme is inhibited by 50%. Three independent experiments were performed in triplicate.

### Development of a NanoBiT-Based Conformational Sensor for SARS-CoV-2 Monomeric M^pro^.

The LgBiT and SmBiT were fused to the N and C termini of monomeric M^pro^, respectively. The expression construct of LgBiT-M^pro^-SmBiT was subcloned in to pGEX4T-1 vector, which coded the LgBiT-M^pro^-SmBiT with the GST-tag in the N-terminal, and then expressed with the *E. coli* BL21(DE3), purified by GST-column, and removed the GST-tag by thrombin enzyme.

The complement luminescence of NanoBiT system has previously been described (5, 25, 26). In brief, in a 100-μL reaction system, 10 nM LgBiT-M^pro^-SmBiT and different nanobodies are mixed and added into a black half-area 96-well plate; the concentration of the nanobodies ranges from 0.02 nM to 2.5 μM. After gently shaking and mixing, incubating at 4 °C for 30 min, adding 10 μM furimazine (GlpBio, Cat#: GC32913) to initiate reaction, the data are processed by GraphPad Prism7 software. All experiments were performed in triplicate.

## Supplementary Material

Supplementary File

## Data Availability

The atomic coordinates have been deposited in the Protein Data Bank (PDB ID codes 7VFA and 7VFB).
