# Supplementary material for: An extended conformation of SARS-CoV-2 main protease reveals allosteric targets
Source: Proc Natl Acad Sci U S A. 2022 Mar 24;119(15):e2120913119. doi: 10.1073/pnas.2120913119 (PMC9169858; doi:10.1073/pnas.2120913119)
Supplement: Supplementary File [file pnas.2120913119.sapp.pdf]

Supplement figures

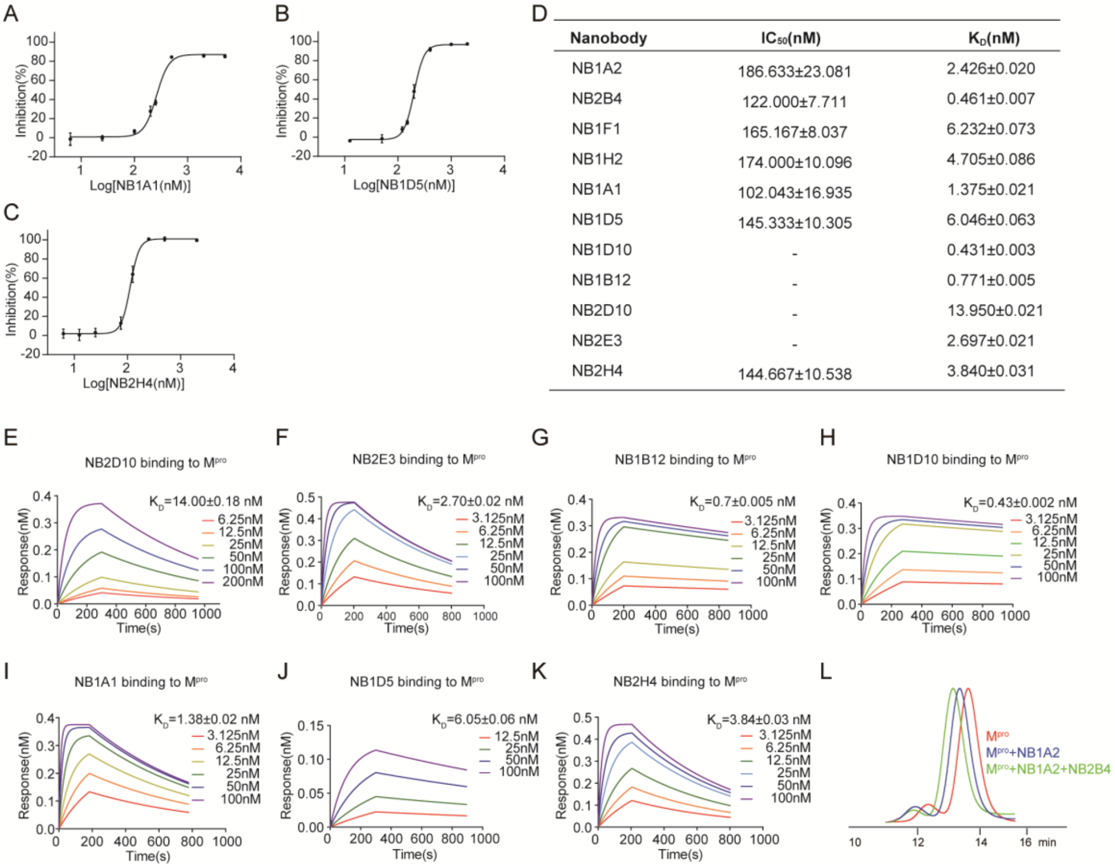

Nanobody	IC50(nM)	KD(nM)
NB1A2	186.633±23.081	2.426±0.020
NB2B4	122.000±7.711	0.461±0.007
NB1F1	165.167±8.037	6.232±0.073
NB1H2	174.000±10.096	4.705±0.086
NB1A1	102.043±16.935	1.375±0.021
NB1D5	145.333±10.305	6.046±0.063
NB1D10	-	0.431±0.003
NB1B12	-	0.771±0.005
NB2D10	-	13.950±0.021
NB2E3	-	2.697±0.021
NB2H4	144.667±10.538	3.840±0.031

Fig. S1. Generation and characterization of camelid inhibitory nanobodies against SARS-CoV-2 M^{pro}. (A-C) The hydrolytic activity of SARS-CoV-2 M^{pro} was measured in the presence of increasing concentrations of the inhibitory nanobodies, NB1A1(A), NB1D5(B), and NB2H4(C). (D) Summary table of the inhibitory effect and the binding affinity of tested NBs to SARS-CoV-2 M^{pro}. (E-K) Biolayer Interferometry (BLI) binding kinetics measurement for NB2D10(E), NB2E3 (F), NB1B12 (G), NB1D10(H), NB1A1(I), NB1D5 (J) and NB2H4 (K) (K_D, equilibrium dissociation constant). (L) Representation of SEC profiling of the monomeric M^{pro} (red), M^{pro}+NB1A2 complex (blue), and M^{pro}+NB1A2+NB2B4 complex (green).

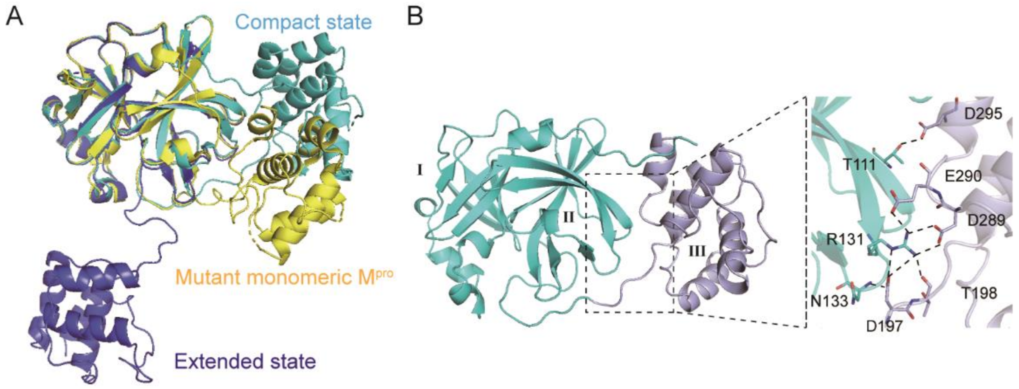

Fig. S2. Two transient conformations of SARS-CoV-2 monomeric M^{pro} are captured by NB2B4 and NB1A2. (A) Superposition of the extended (blue), the compact (cyan) and mutant (G11A, PDB: 2pwx) monomeric M^{pro} (yellow) based on the catalytic domain. (B) the interface between the N-terminal domain (cyan) and C-terminal domain (light blue) in monomeric compact structure (left panel), Close-up views of the interaction between N-terminal domain (cyan) and C-terminal domain (light blue) (right panel). The key residues involved in interaction are shown as stick models. Polar interactions are indicated with black dashed lines.

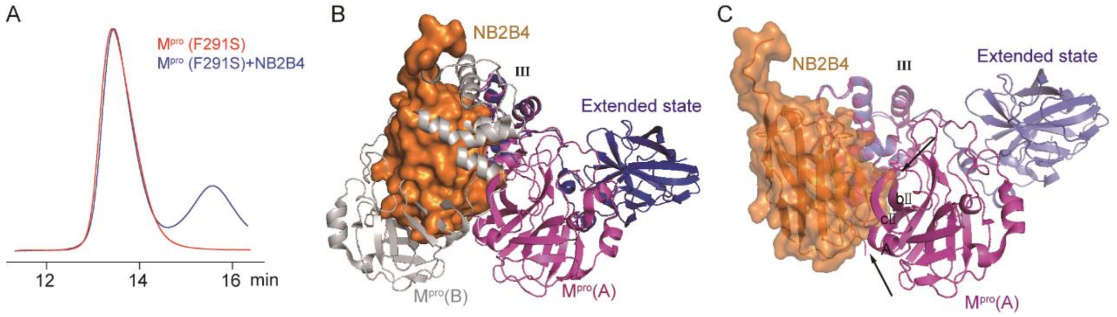

Fig. S3. Structural mechanism of inhibition of SARS-CoV-2 M^{pro} by NB2B4.(A) Representation of SEC profiling of the mutant (F291S) monomeric M^{pro} (red), and mutant (F291S) monomeric M^{pro}+NB2B4 complex (blue). The peak of mutant monomer overlaps the peak of the mixture of the mutant monomer and NB2B4, indicating that the mixture doesn't form the complex. (B-C) Superposition of the extended (blue) monomeric M^{pro} with NB2B4 (electrostatic surface, orange) and one protomer (A protomer magenta) of dimeric M^{pro} based on the C-terminal (B) and the N-terminal (C), another protomer (B protomer, gray). It indicates that the NB2B4 (electrostatic surface, orange) has steric clash with the catalytic domain of A protomer and the whole structure of B protomer.

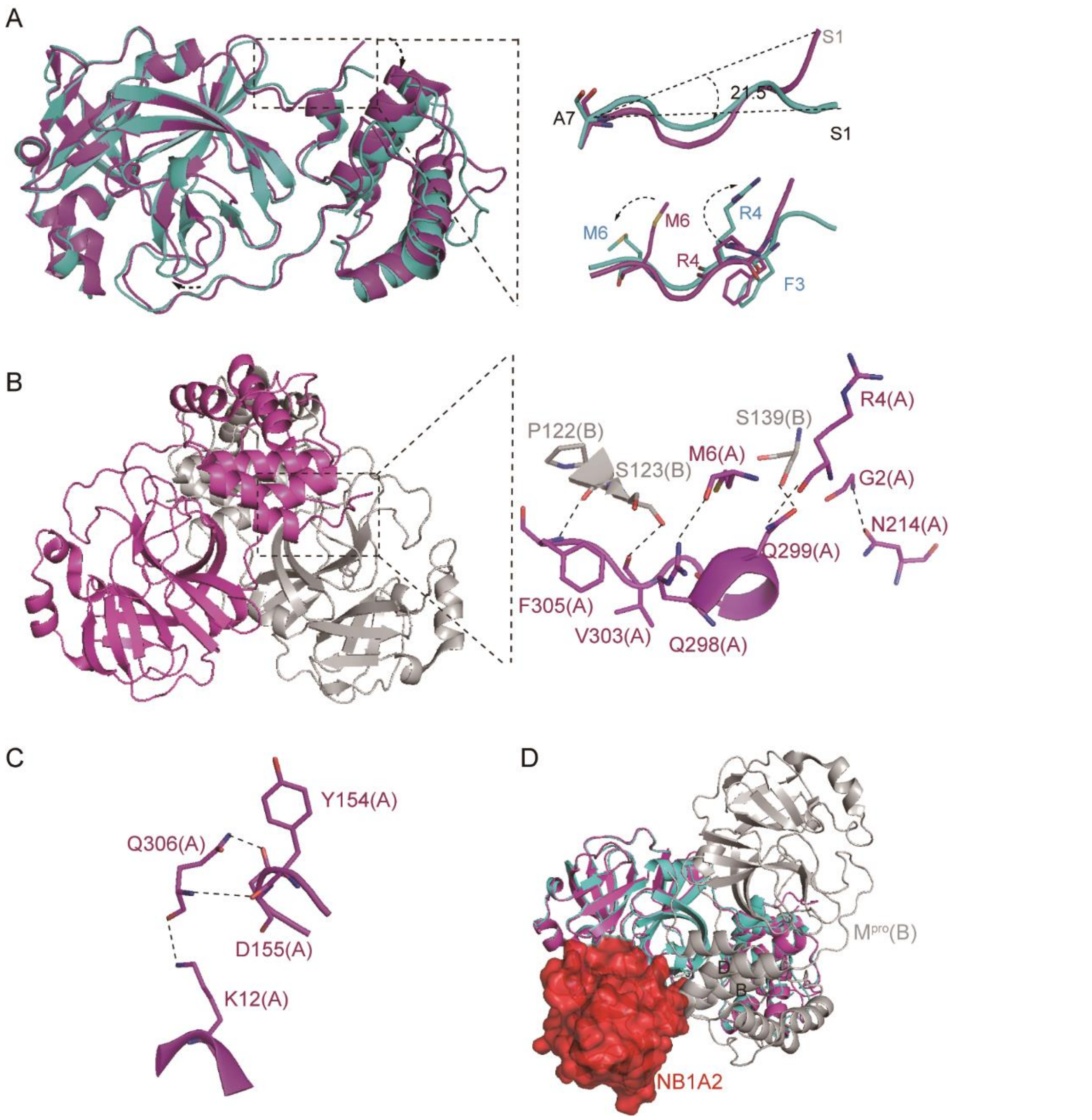

Fig. S4. Structural mechanism of inhibition of SARS-CoV-2 M^{pro} by NB1A2. (A) The structural conformational changes of the N-finger in the compact state (cyan) of M^{pro}, compared with that of active dimeric M^{pro} (magenta). Superposition of the compact state (cyan) and a protomer of active dimeric M^{pro} (magenta) (left panel). The N-finger in the compact state rotate about 21.5^{o}, compared to the active dimeric M^{pro} (right-up panel). The dislocation of the key residues F3, R4 and M6 (shown as stick models) of N-finger of compact monomeric state (cyan), compared to the active dimeric M^{pro} (magenta) (right-down panel). (B-C) The orientation of C-terminal residues is fixed by the dimerization of M^{pro}. (B) The interaction between the C-terminal peptide from one protomer (A protomer, colored in magenta) and another protomer (B protomer, colored in gray) (left panel), close-up views of the interface between the C-terminal peptide from one protomer (A protomer, colored in magenta) and another protomer (B protomer, colored in gray) (right panel). (C) the last residue Q306 of the C-terminal domain interacts with the residues of N-terminal catalytic domain. (D) Superposition

of the compact (cyan) monomeric M^{pro} with NB1A2 (electrostatic surface, red) and one protomer (A protomer magenta) of dimeric M^{pro} based on the C-terminal domain, another protomer (B protomer, pink). It indicates that the NB1A2 (electrostatic surface, red) has steric clash with the C-terminal domain of B protomer.

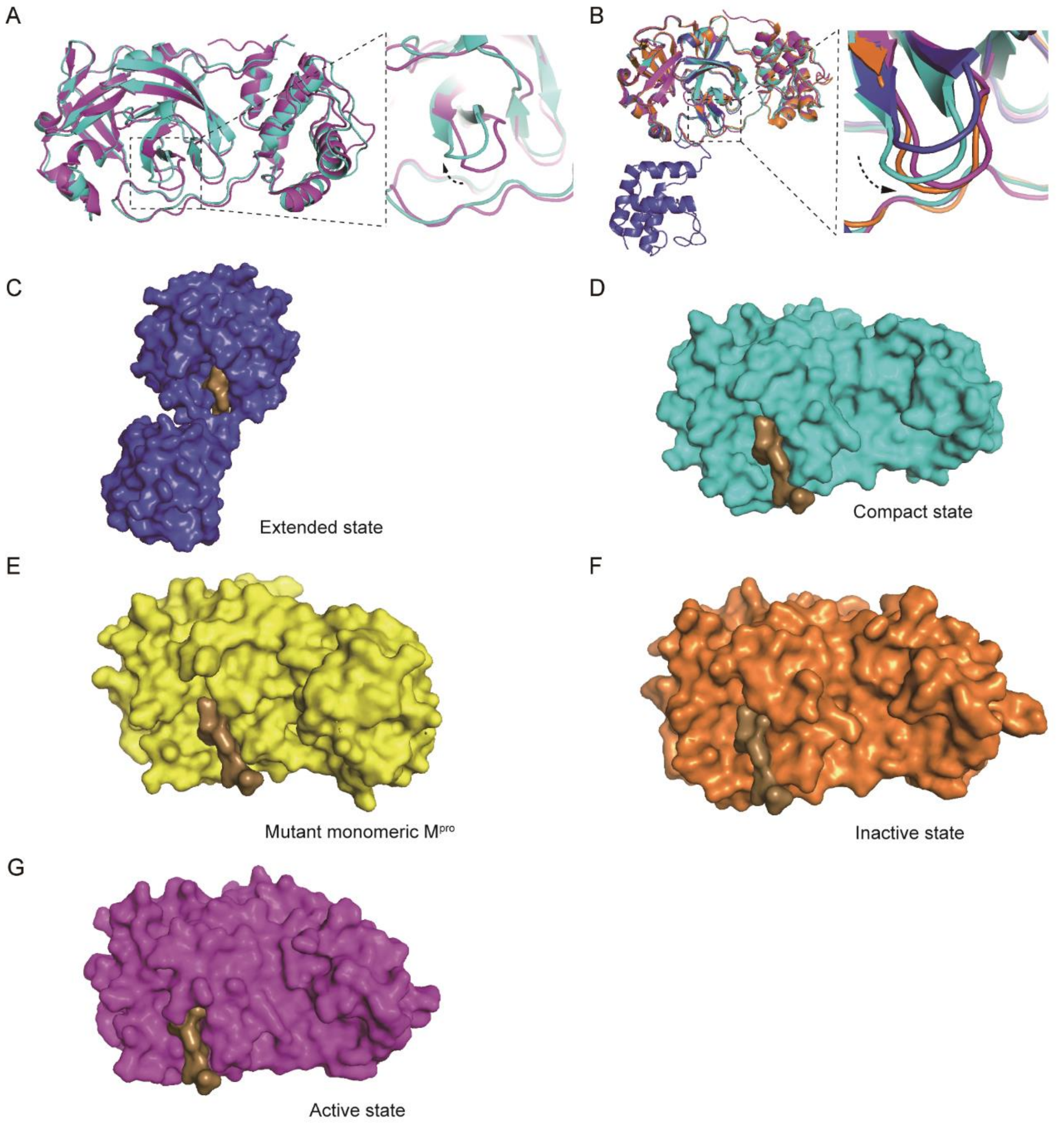

Fig. S5. The conformation of monomeric M^{pro} is incompatible with the substrate binding. (A) Superposition of the compact monomeric structure and one protomer of dimeric structure based on the N-terminal domain, with the compact structure colored in cyan, and dimer structure colored in purple (left panel), close-up view of the conformational change of β-turn (right panel). (B) Superposition of the extended (blue), compact (cyan), and G11A mutant (yellow) monomeric structures, and inactive (orange) and active (magenta) protomer of dimeric structure based on the N-terminal domain (left panel), close-up view of the conformational change of β-turn (right panel). (C-G) The model of different M^{pro} state and substrate (TSAVLQ, derived from the N-terminal autocleavage sequence of the viral protease), the volume of substrate bound pocket in

different state except for active protomer is insufficient to accommodate the substrate. The M^{pro} and substrate are shown as surface and color-coded as in B.

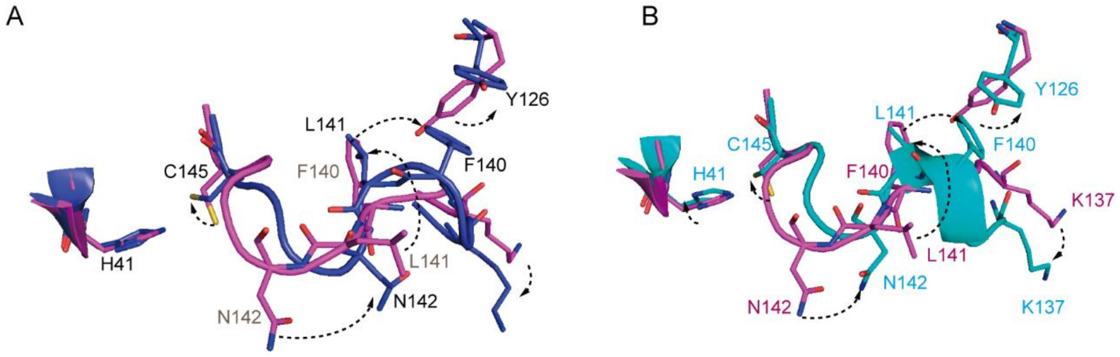

Fig. S6. The conformational changes of the active loop in the extended and compact monomeric state, compared with the active loop conformation of dimeric M^{pro}. The structural conformational changes of the active loop in the monomeric extended state (A, blue) and the monomeric compact state (B, cyan) of M^{pro}, compared with that of active dimeric M^{pro} (magenta), The residues are shown as stick models.

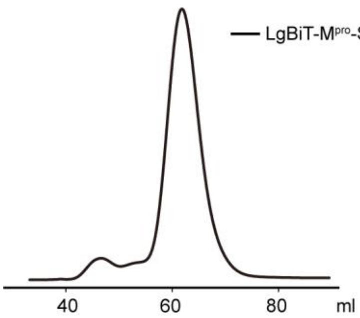

Fig. S7. Representation of SEC profile of the monomeric LgBiT-M^{pro}-SmBiT. The LgBiT and SmBiT were fused to the N-termini and C-termini of monomeric M^{pro}, respectively. The LgBiT-M^{pro}-SmBiT was purified by GST-column, and polished by SEC.

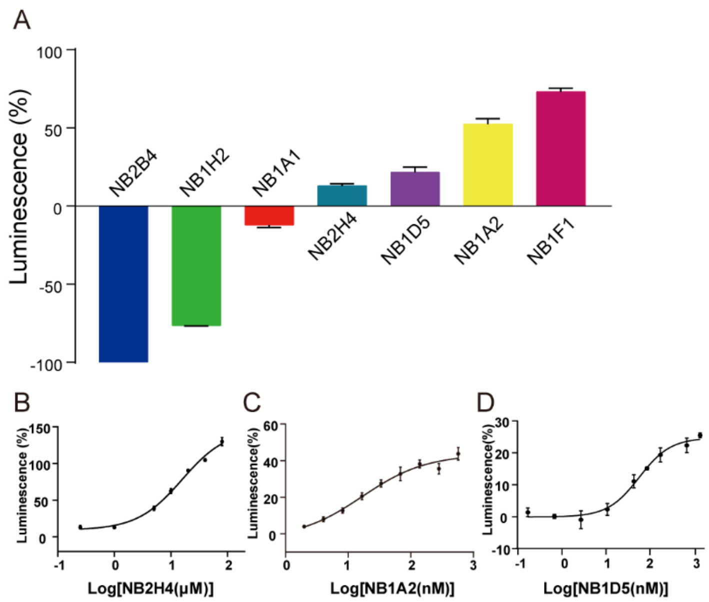

Fig. S8. The percent change of luminescence signal in the presence of different nanobodies (the concentration is 1μM). (A) The results showed that in the presence of NB2B4 or NB1H2 or NB1A1, the luminescence decreased, while in the presence of NB2H4 or NB1D5 or NB1A2 or NB1F1, the luminescence signal increases, and the effects are different. Luminescence signal measured for NB2H4 (B), NB1A2(C), NB1D5(D) in different concentrations with the pEC_{50} values about 15.680 μM, 15.760 nM and 41.260 nM, respectively. Data points represent mean ± SEM of triplicate measurements.
